# Gammaherpesvirus Infection Stimulates Lung Tumor-Promoting Inflammation

**DOI:** 10.3390/pathogens13090747

**Published:** 2024-08-31

**Authors:** Sudurika S. Mukhopadhyay, Kenneth F. Swan, Gabriella Pridjian, Jay K. Kolls, Yan Zhuang, Qinyan Yin, Joseph A. Lasky, Erik Flemington, Cindy A. Morris, Zhen Lin, Gilbert F. Morris

**Affiliations:** 1 Departments of Microbiology & Immunology and Pathology & Laboratory Medicine, School of Medicine, Tulane University, New Orleans, LA 70118, USA; smukhopa@tulane.edu; 2 Department of Obstetrics & Gynecology, School of Medicine, Tulane University, New Orleans, LA 70118, USA; kswan1@tulane.edu (K.F.S.); pridjian@tulane.edu (G.P.); 3 Departments of Medicine & Pediatrics, School of Medicine, Tulane University, New Orleans, LA 70118, USA; jkolls1@tulane.edu; 4 Division of Pulmonary, Critical Care and Environmental Medicine, Department of Medicine, School of Medicine, Tulane University, New Orleans, LA 70118, USA; yzhuang1@tulane.edu (Y.Z.); qyin@tulane.edu (Q.Y.); jlasky@tulane.edu (J.A.L.); 5 Department of Pathology & Laboratory Medicine, School of Medicine, Tulane Cancer Center, Tulane University, New Orleans, LA 70118, USA; erik@tulane.edu (E.F.); zlin@tulane.edu (Z.L.); 6 Department of Microbiology and Immunology, School of Medicine, Tulane University, New Orleans, LA 70118, USA; cmorris2@tulane.edu

**Keywords:** γherpesvirus, EBV, MHV68, MDSCs, tumorigenesis, Type 17 inflammation, IL-17, IL-6, CSF3, CXCL1, m^6^A methylation, K-Ras^LA1^

## Abstract

Lung tumor-promoting environmental exposures and γherpesvirus infections are associated with Type 17 inflammation. To test the effect of γherpesvirus infection in promoting lung tumorigenesis, we infected mutant K-Ras-expressing (K-Ras^LA1^) mice with the murine γherpesvirus MHV68 via oropharyngeal aspiration. After 7 weeks, the infected mice displayed a more than 2-fold increase in lung tumors relative to their K-Ras^LA1^ uninfected littermates. Assessment of cytokines in the lung revealed that expression of Type 17 cytokines (*Il-6*, *Cxcl1*, *Csf3*) peaked at day 7 post-infection. These observations correlated with the post-infection appearance of known immune mediators of tumor promotion via IL-17A in the lungs of tumor-bearing mice. Surprisingly, *Cd84*, an immune cell marker mRNA, did not increase in MHV68-infected wild-type mice lacking lung tumors. Csf3 and Cxcl1 protein levels increased more in the lungs of infected K-Ras^LA1^ mice relative to infected wild-type littermates. Flow cytometric and transcriptomic analyses indicated that the infected K-Ras^LA1^ mice had increased Ly6G^dim^/Ly6C^hi^ immune cells in the lung relative to levels seen in uninfected control K-Ras^LA1^ mice. Selective methylation of adenosines (m^6^A modification) in immune-cell-enriched mRNAs appeared to correlate with inflammatory infiltrates in the lung. These observations implicate γherpesvirus infection in lung tumor promotion and selective accumulation of immune cells in the lung that appears to be associated with m^6^A modification of mRNAs in those cells.

## 1. Introduction

Lung cancer is the leading cause of cancer-related mortality worldwide, comprising almost 25% of all cancer-related deaths [1,2]. In the US, the primary environmental risk factor for lung cancer is tobacco smoke [3]. Human lung carcinogens such as tobacco smoke, asbestos, and silica elicit Type 17 inflammation, an inflammatory phenotype that induces tumorigenesis in autochthonous murine lung tumor models [4,5,6,7]. IL-17A (typically referred to as IL-17 and will be so hereafter), the hallmark cytokine of Type 17 inflammation, can be expressed by a variety of innate and adaptive immune cell types, but CD4+ T helper cells are the principal source [8]. The significance of the relationship between Type 17 inflammation and lung carcinogenesis has been heightened by the recent observation that normal human lung tissue displays a high incidence (>50%) of lung tumor-promoting oncogenes [9]. These observations are consistent with the concept that the chronic inflammation induced by environmental exposures, more specifically chronic Type 17 inflammation, contributes to the pathogenesis of lung cancer. Thus, other agents that trigger Type 17 inflammation could contribute to human lung cancer.

The observation that the genomes of two γherpesviruses, Herpesvirus saimiri and Kaposi’s sarcoma herpesvirus (KSHV), encode the Type 17 cytokines IL-17 and IL-6 [10,11], respectively, suggests that Type 17 inflammation is integral to γherpesvirus replication. The association of γherpesviruses with Type 17 inflammation suggests that these viruses, particularly Epstein–Barr virus (EBV) in humans, could be contributors to lung tumorigenesis [10,12,13,14]. The γherpesviruses are host specific; therefore, the similar murine γherpesvirus 68 (MHV68) has generally served as a genetically and biologically relevant model for in vivo interactions between the γherpesviruses and their host [15]. Like EBV, intranasal infection of mice with MHV68 leads to productive infection of cells of the respiratory mucosa and viral persistence in a variety of immune cell types, primarily B-lymphocytes. Plasma cell differentiation of latently infected B cells correlates with virus reactivation from latency [16]. The pathology induced by the γherpesvirus infection/reactivation is generally the consequence of T cell proliferation to repress virus-infected B cells [17].

Although it is estimated that EBV infects more than 90% of the world’s population [18], only a small fraction (~1.5%) of human cancers worldwide express EBV viral transcripts in the tumor cells [19]. EBV contributes to neoplastic and lymphoproliferative disorders including undifferentiated nasopharyngeal carcinoma, lymphomas, gastric carcinomas, and breast cancer [19,20,21], but its association with lung cancer has been less defined [22,23,24]. The ability of the virus to undergo latency and persist long term in memory B cells [19] provides a means to perpetuate chronic effects that benefit virus propagation and coincidentally favor tumorigenesis. Although not considered a major lung carcinogen, EBV has been suspected to play a role in exacerbating lung cancer [23,24]. In addition to the mode of γherpesvirus-related tumorigenesis in the tumor types cited above, the data shown here are consistent with the concept that virus infection gives rise to lung tumor-promoting inflammation, which includes lung accumulation of immune cells with characteristics previously identified as pro-tumorigenic in lung cancer [25].

## 2. Materials and Methods

### 2.1. MHV68 Preparation

BALB/3T12-3 cells were used to grow MHV68, whereas BHK-21/C-13 cells were used for viral titration. Both cell lines were purchased from ATCC and cultured in complete Dulbecco’s modified Eagle’s medium (DMEM with glutamax) supplemented with 10% fetal bovine serum (FBS), 100 U of penicillin/mL, and 100 mg of streptomycin/mL [26,27]. BALB/3T12-3 cells were passaged into 5–15 cm plates and grown until they reached 80–90% confluence. BALB/3T12-3 cells were infected with MHV68 (ATCC) at an MOI of 0.1 in DMEM (2% FBS, 1% penicillin/streptomycin) and incubated at 37.0 °C in the cell culture incubator and gently rocked at 15 min intervals. After 2 h, the media of the infected cells was replaced with 10% FBS and the cells were further incubated for another 2–8 days [28]. Once cytopathic effects (CPEs) were observed (75–80%), the incubation was terminated and the cells were collected by scraping and transferred into a 50 mL sterile tube with associated media and stored at −80 °C [29,30]. Cell lysis (mechanical disruption) was induced by freeze–thawing the cells 3 times, completely inverting tubes, and pipetting up and down multiple times. The cell lysate was centrifuged at 2800× *g* for 20 min at 16.0 °C to remove cell debris. The lysate was then layered over a thin layer of 20% sorbitol in an ultracentrifuge tube followed by ultracentrifugation at 22,000 rpm for 2 h and 40 min at 4.0 °C. The pellet (purified virus) was resuspended in 4 mL of PBS, aliquoted (200 µL) into vials, and stored in liquid nitrogen. MHV68 titration was performed in BHK-21/C-13 cells using 6-well plates. Cells were seeded at 150,000 cells/well in DMEM (10% FBS, 1% L-glutamine) and infected the next day at 50–70% confluency. Each viral dilution was performed in triplicate. An uninfected control and 6 serial 10-fold dilutions (10^−3^, 10^−4^, 10^−5^, 10^−6^, 10^−7^, 10^−8^) were included in the titration. The 1 h incubation was interrupted every 10–15 min to rock the plates gently. Upon completion, each well was overlaid with 4 mL of warm methyl cellulose (Sigma-Aldrich, St. Louis, MO, USA) in DMEM with glutamax (2.5% calf serum, 100 U of penicillin/mL, 100 mg of streptomycin/mL) and incubated a further 4 to 5 days at 37.0 °C. Once plaques were visible, the methyl cellulose was aspirated and the cells were stained with 0.1% crystal violet (Sigma) and then rocked on a plate shaker for 30 min at room temperature. The wells were washed with purified H_2_O and air dried. Plaques were counted with the naked eye and counts were later confirmed using an inverted microscope [27,28]. The titer was calculated using counts from 2 dilutions. Each count was an average of triplicate readings.

### 2.2. Animal Model

The National Cancer Institute Mouse Repository provided K-Ras^LA1^ mice, which were crossed into the C57BL/6 (B6) background more than 10 times. Experimental protocols were approved by the Tulane University Institutional Animal Care and Use Committee in agreement with the National Institutes of Health guidelines provided by the Association for Assessment and Accreditation of Laboratory Animal Care. Mice were maintained under select pathogen-free conditions and mating pairs were set up for the procurement of WT and K-Ras^LA1^ genotypes. Mouse chow and water were provided ad libitum. Littermates were infected with virus between 3.5 and 5 weeks of age with 40,000 plaque forming units (pfu) of MHV68 diluted in a 50 µL PBS solution. Control mice were treated with an equivalent volume of PBS. The anesthesia of mice during treatment was accomplished using isoflurane (VetOne, Boise, ID, USA), where its influx into a confined chamber for 15–25 s ensured loss of consciousness. Mice were subsequently hung vertically with a silk thread behind their front teeth. Pinching the nose and pulling the tongue forward allowed for viral delivery via oropharyngeal aspiration [4]. Infected and uninfected mice resided in separate microisolators until harvesting. Euthanasia of the mice was accomplished by intraperitoneal delivery of 0.8 mg/kg avertin (ThermoFisher, Waltham, MA, USA) followed by exsanguination. Cardiac puncture was performed using a 3 mL syringe targeting the ventricle of the heart. Approximately 0.5–1.0 mL of blood was collected and allowed to coagulate before centrifugation at 1500× *g* for 10 min. The renal artery was severed to allow for complete exsanguination before tissue collection.

### 2.3. RNA Preparation and Analysis

The spleen was collected for RNA extraction. The collection and processing of total lung tissue differed depending on the assay. Total RNA was prepared from the left lung for the time course (3–9 days post-infection, DPI) experiments. The left lung was removed, placed in bead tubes (ThermoFisher, Waltham, MA, USA), and homogenized for 1 min at 5 m/s in a Beadmill (ThermoFisher, Waltham, MA, USA) in 1 mL Trizol. The right lung was reserved for protein lysates by removal to cryovials before freezing in liquid nitrogen. For the tumor correlation assay (7 DPI), the left bronchus was ligated and the left lung was processed for RNA extraction as above. The right lung was inflated and fixed with formalin for tumor analysis. For the tumor promotion assay (7-weeks PI), both lungs were inflated and fixed with formalin for tumor analyses [4]. During lung inflation, lung(s) were inflated at 30cm pressure, fixed with formalin for 20 min, then stored at 4 °C in formalin overnight and transferred to PBS before paraffin embedding. Tail snips were collected to confirm genotypes. RNA was extracted using the RNeasy Mini Kit as described by the supplier (Qiagen, Germantown, MD, USA) [4]. Total RNA samples were Dnased using the Turbo DNA-free Kit (Invitrogen, Carlsbad, CA, USA) and then underwent cDNA Synthesis using the iScript cDNA Synthesis Kit (Bio-Rad, Hercules, CA, USA) [31]. A NanoDrop Spectrophotometer (ThermoFisher, Waltham, MA, USA) was used to quantify RNA. 

### 2.4. Reverse Transcription-Quantitative Polymerase Chain Reaction (RT-qPCR)

RT-qPCR was carried out in 10 µL using Sso Advanced Universal SYBR Green Supermix (Bio-Rad). A mastermix was prepared for each primer set and loaded onto a 96-well plate along with the designated samples. Thermal cycling was run on a CFX Opus 96 Real-Time PCR System (Bio-Rad) using an optimized protocol: 95.0 °C for 3 min; 95.0 °C for 10 s; annealing temperature (usually 56.0 °C) for 30 sec; initial plate reading; return to step 2–39 times; melt curve 65.0 °C to 95.0 °C; increment 0.5 °C for 5 s; second plate reading. Dissociation (melting) curve analyses were performed. Quantification was performed using the 2^-DDCt^ method, normalizing all CT scores to beta-actin (ß-actin). Final measurements were in fold change normalized to PBS-treated mice harvested on day 7 for the time course and tumor correlation assays. *Orf65* mRNA levels were normalized to MHV68-infected mice on day 7 post-infection in the time course assay and in the tumor correlation assay. Table 1 presents the quantitative PCR mouse target genes with primer sets.

### 2.5. Protein Extraction of Mouse Lung Tissue

Protein was extracted from mouse lung tissue for ELISA assays. The protocol enlisted was optimized for the homogenization of protein using a Bead Mill 4 (ThermoFisher, Waltham, MA, USA). Samples were incubated in a 1:100 dilution of protease inhibitor cocktail (100×) (Cell Signaling Technology, Danvers, MA, USA) in PBS and homogenized using the Bead Mill for 30 s at a power of 5 m/s. RIPA buffer (Cell Signaling, Danvers, MA, USA) was added to a final 1× concentration and samples were vortexed briefly. Samples were centrifuged for 15 min at 20,817× *g* at 4.0 °C and the supernatant was subsequently stored at −80.0 °C. Lysates were quantified for protein concentration using the BCA Assay (ThermoFisher, Waltham, MA, USA).

### 2.6. Enzyme-Linked Immunosorbent Assay (ELISA)

Lung lysates and serum were used to quantify the protein levels of Csf3, Cxcl1, and Il-6 using ELISA. Detection of Csf3 and Cxcl1 was accomplished using Mouse CSF3/G-CSF and CXCL1/KC Immunoassay kits according to the supplier’s instructions (Quantikine, Minneapolis, MN, USA). The ELISA for Il-6 was carried out using the LEGEND MAX^TM^ Mouse IL-6 ELISA Kit according to the supplier’s instructions (BioLegend, San Diego, CA, USA).

### 2.7. Tumor Counting

Pleural surface tumor counts on fixed inflated lung tissue was performed by 3 individuals independently while blinded to the sample’s identity. These counts were normalized for each individual’s average tumor counts of the PBS-treated mice. For tumor lesion counts, H&E-stained slides prepared from paraffin-embedded lung tissue were quantified for proliferative lesions by light microscopy in a blinded manner.

### 2.8. Immunofluorescence

Formalin-fixed paraffin-embedded lung tissue sections were co-stained with Ly6G and IL-6 antibodies in an immunofluorescence assay (Table 2). All incubations were performed in a humid chamber and incubation solutions were sterile filtered. Slides were heated overnight at 50.0 °C, deparaffinized in xylene, and hydrated in graded alcohol solutions. Antigen retrieval was performed by heating the slide in 10 mM Sodium Citrate for 20 min by bringing the solution to a boil (100.0 °C) with intervals of cooling. All washes were performed with 1x Dulbecco’s phosphate-buffered saline (DPBS) on a rocker prior to antibody incubations, whereas 1XDPBST with 0.1% tween was used for post-antibody washes. The samples were blocked with 1XDPBS 10% normal goat serum (NGS) for 1 h at room temperature and antibody dilutions were made in 1XDPBS with 2% NGS. The protocol was a sequential stain of 1 µg of Purified Rat anti-Mouse Ly6G (BD Pharmingen, San Diego, CA, USA), followed by a 1:150 dilution of IL-6 Antibody (Novus Biologicals, Centennial, CO, USA), and incubation overnight in 4.0 °C. The secondary antibodies for Ly6G and IL-6 were a 1:200 Goat Anti-Rat IgG H&L (Alexa Fluor 647) (Abcam, Waltham, MA, USA) and a 1:1000 Goat Anti-Rabbit IgG H&L (Alexa Fluor 488) (Abcam), respectively, which were incubated in the dark at room temperature for 1 h. Slides were mounted using ProLong Gold antifade reagent with DAPI (Invitrogen, Carlsbad, CA, USA). An inverted Nikon microscope was used for detection at 600× magnification.

### 2.9. Flow Cytometry

Flow cytometry was used to quantify MDSCs. Cells underwent single-cell isolation using a standard protocol designed for “Lung Harvest and Digestion for Flow Cytometry” [32]. Lung tissue was minced and digested with collagenase from clostridium histolyticum (Sigma) at a final concentration of 1 mg/mL. Cells were incubated horizontally on a shaker at 37.0 °C for 1 h on moderate-to-low speed and were then filtered through a 70-micron filter and centrifuged at 500× *g* for 5 min at 4.0 °C. The pellet was resuspended in RPMI media supplemented with 10% newborn calf serum (NCS), 100 U of penicillin/mL, and 100 mg of streptomycin/mL and counted in preparation for cell sorting. Cells were selected for Gr-1^-^ myeloid cells using the Myeloid-Derived Suppressor Cell Isolation Kit (Miltenyi Biotec, Gaithersburg, MD, USA). Selected Ly6G-positive cells were stained with APC-eFluor780-CD11b (ThermoFisher, Waltham, MA, USA), APC anti-mouse Ly6C (BioLegend), V500 Mouse anti-Mouse CD45.2 (BD Horizon, Franklin Lakes, NJ, USA), and PE Rat Anti-Mouse Ly6G (BD Pharmingen) antibodies. The final concentration for all antibodies in solution was 0.01 µg/µL or 1 µg. An Fc-receptor blocking antibody TruStain FcX^TM^ (anti-mouse CD16/32) Antibody (BioLegend, San Diego, CA, USA) (2.5 µg) and True-Stain Monocyte Blocker (1:20 dilution) were added to the staining solution. After incubation, the cells were washed with PBS and fixed in 1% formaldehyde PBS solution in preparation for flow cytometry [27,32]. Samples were processed via flow cytometry at the Microbiology/Immunology Core facility, a shared resource lab, whereby detection of fluorescently labeled cells passing through a cytometer identified Gr-1^+^ cells. Positive selection with anti-Gr1 antibodies in the flow through fraction from the Ly6G selection identified Ly6G^dim^ Ly6C^hi^ cells.

### 2.10. Immunoprecipitation of Methylated mRNAs (m^6^A Me-RIP Assay)

Total RNA (70 µg) prepared from mouse lung tissue was submitted to LC Sciences for m^6^A immunoprecipitation and RNA sequencing. After RNA isolation and DNasing, as described above, the samples were subjected to additional purification using the RNeasy Mini Kit (Qiagen) prior to submission [33]. The processing of samples involved RNA fragmentation, RNA immunoprecipitation (RIP), and elution of m^6^A-modified transcripts as determined by the service provider (LC Sciences, Houston, TX, USA). Eluted m^6^A RNAs and input ribosomal-depleted RNA samples were sequenced at ~40 million 150 bp paired-end reads.

### 2.11. Statistical Analysis

Unless indicated otherwise, the data are reported as mean +/− SEM and significance is determined via Mann–Whitney two-tailed t-tests using GraphPad Prism software version 10 for statistical analysis. All data are normalized to the uninfected control mice. RT-qPCR data are normalized to ß-actin.

## 3. Results

### 3.1. MHV68 Induces Type 17 Cytokines during Virus Infection

**Figure 1:**
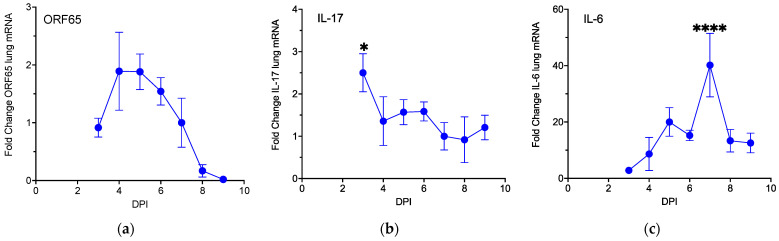
MHV68 infection activates expression of Type 17 cytokines. The graphs represent the mean fold change +/- SEM relative to the control for the indicated mRNA (*n* = 3–9 infected; *n* = 13 control) versus the days post-infection (DPI) for mRNAs encoding: (**a**) *Orf65,* normalized to the levels detected on day 7, (**b**) *Il-17* (* *p* = 0.0205), and (**c**) *Il-6* (**** *p <* 0.0001 uninfected versus MHV68 infected). *Il-17* and *Il-6* in MHV68-infected wild-type (WT) mice are normalized to PBS-treated control WT mice at day 7.

Since recent findings link Type 17 inflammation with MHV68 replication [12,13], we set out to test if MHV68 infection could accelerate tumorigenesis in lung-tumor-bearing K-Ras^LA1^ mice. An initial experiment with increasing doses of virus demonstrated that 40,000 pfu of MHV68 elicited maximal *Orf65* mRNA expression, a late transcript encoding a component of the viral capsid that is used here to monitor viral replication. This dose of virus elicited modest lung inflammation and no overt adverse effects on the mice in the form of lethargy or weight loss. Subsequently, to establish the relationship between cytokine production and viral infection, wild-type mice infected with 40,000 pfu MHV68 were harvested daily between days 3 and 9 post-infection and RNA was extracted from the lung tissues of the infected mice for RT-qPCR analysis. Mock-infected mice at day 7 were used as the negative control group. Serum and lung tissue lysates were also prepared for protein quantification by ELISAs. The levels of *Orf65* mRNA in the lungs of MHV68-infected wild-type mice had increased by day 3 post-infection and diminished to near undetectable levels by day 9 (Figure 1a). The levels of *Il-17* mRNA decreased ~2.5-fold from a high at day 3 to low levels at days 7 through 9 in MHV68-infected mice (Figure 1b). The most pronounced effect was observed for the mRNA levels of *Il-6*, which peaked on day 7 post-infection with a 40-fold increase relative to mock-infected mice (Figure 1c). Induction of IL-6 in MHV68-infected wild-type mice was further confirmed via ELISA assays for Il-6 in the serum and lung lysates, which revealed 1.8-fold and 9.4-fold increases, respectively, relative to mock-infected mice (Figure S1). These observations are consistent with induction of a Type 17 cytokine response to MHV68 infection.

### 3.2. MHV68 Infection Promotes Proliferative Lesions in Tumor-Bearing Mice

**Figure 2:**
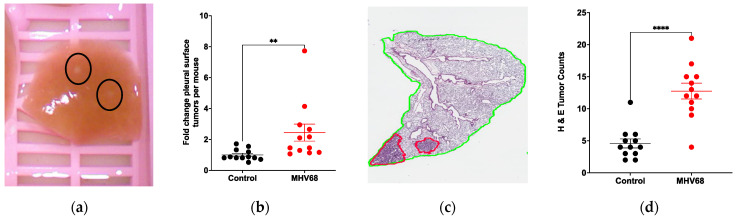
MHV68 infection induces proliferative lesions in K-Ras^LA1^ mice. (**a**) Tumor nodules on the pleural surface of fixed lung tissue. (**b**) The tumor nodules from uninfected and MHV68-infected K-Ras^LA1^ mice were counted by 3 individuals unaware of the sample identity. The individual values for each mouse were normalized to the mean of the control for each observer. Each symbol represents the fold change of the mean number tumors from the 3 assessments of each mouse (** *p* = 0.0013 mock-infected versus MHV68-infected). (**c**) The fixed lung tissues from the mice in panel b were paraffin-embedded prior to preparation of tissue sections, which were H&E stained to reveal histopathology. Proliferative lung lesions (encircled red) were identified in a blinded manner. (**d**) Proliferative lung lesions were totaled for each mouse. The graph shows the number of proliferative lesions per section for each mouse. (**** *p* < 0.0001 mock infected versus MHV68 infected).

We next tested if MHV68 infection enhanced lung tumorigenesis. K-Ras^LA1^ mice were infected with 40,000 pfu of MHV68 and control K-Ras^LA1^ mice were mock-infected with vehicle (PBS). Both groups were harvested 7-weeks post-infection and tumor nodules on the pleural surface of fixed lung tissue were quantified. MHV68-treated K-Ras^LA1^ mice had a 2-fold increase in tumors relative to their PBS-treated littermates (Figure 2a,b). The lung tissues were subsequently paraffin embedded for preparation of tissue sections that were stained with hematoxylin and eosin and assessed microscopically for proliferative lesions. The MHV68-infected mice had an almost a 3-fold increase in lung tumors relative to the mock-infected control group (Figure 2c,d). These analyses confirmed that MHV68 infection promoted tumorigenesis in mutant K-Ras-expressing mice.

### 3.3. Promotion of Inflammation by MHV68 Infection

**Figure 3:**
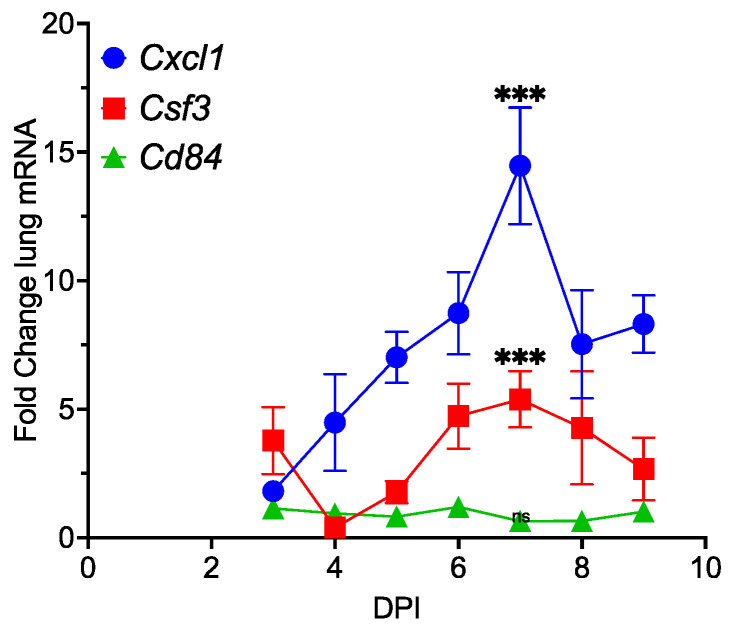
MHV68 infection of wild-type mice elicits cytokines that promote granulocyte recruitment and survival while not affecting expression of *Cd84*, an immune cell marker mRNA. Levels of the mRNAs for *Cxcl1*, *Csf3*, and *Cd84* in the lungs of wild-type mice were determined at increasing times post-infection with MHV68 (see Figure 1). The graph shows the fold-change lung mRNA for *Cxcl1* (blue circles), *Csf3* (red squares), and *Cd84* (green triangles) for the indicated day post-infection (DPI). (*** *p* = 0.001 infected versus uninfected control, *n* = 3–9 infected; *n* = 13 control).

Lung tumor promotion by IL-17 is dependent upon the recruitment of immune cells [5,6]. Injection of an antibody against Gr-1 represses IL17-mediated enhancement of lung tumorigenesis in mutant K-Ras-expressing mice [5,6]. IL-17 upregulates the cytokines Cxcl1 and Csf3, which promote the recruitment and survival of MDSCs. [4,5,34,35,36,37]. Furthermore, mRNAs encoding these cytokines are induced by IL-17 in a murine lung tumor cell line (mK-Ras-LE cells) derived from K-Ras^LA1^ mice (Figure S2). RT-qPCR analyses of total lung RNA at increasing times following the MHV68 infection of WT mice revealed a 5-fold increase in *Csf3* mRNA and a 14-fold increase in *Cxcl1* mRNA, both peaking on day 7 (Figure 3). Thus, mRNAs encoding cytokines involved in immune cell recruitment during lung tumor promotion by IL-17 are increased in MHV68-infected mice at day 7 post-infection.

Single-cell RNA sequencing studies identified *Cd84* mRNA as a novel marker for MDSCs in a murine breast tumor model [34]. Lung RNA from the time course experiment described above was evaluated for *Cd84* mRNA. In contrast to expectation, the analysis revealed little change in lung *Cd84* mRNA levels in MHV68-infected wild-type mice (Figure 3). A time course analysis for an additional MDSC marker identified in the single-cell study, *Jaml* mRNA, revealed variable levels that did not appear to correlate with the cytokine mRNA profile shown in Figure 3 or the days post-infection of wild-type mice (Figure S3). These observations showed that the cytokine mRNAs (Cxcl1 and Csf3) involved in the recruitment and survival of MDSCs are expressed during the MHV68 infection of wild-type mice but that mRNA markers for those cells (*Cd84* and *Jaml*) do not increase in the lung.

### 3.4. Disparate Recruitment of Immune Cells in Wild-Type and K-Ras^LA1^ Mice

**Figure 4:**
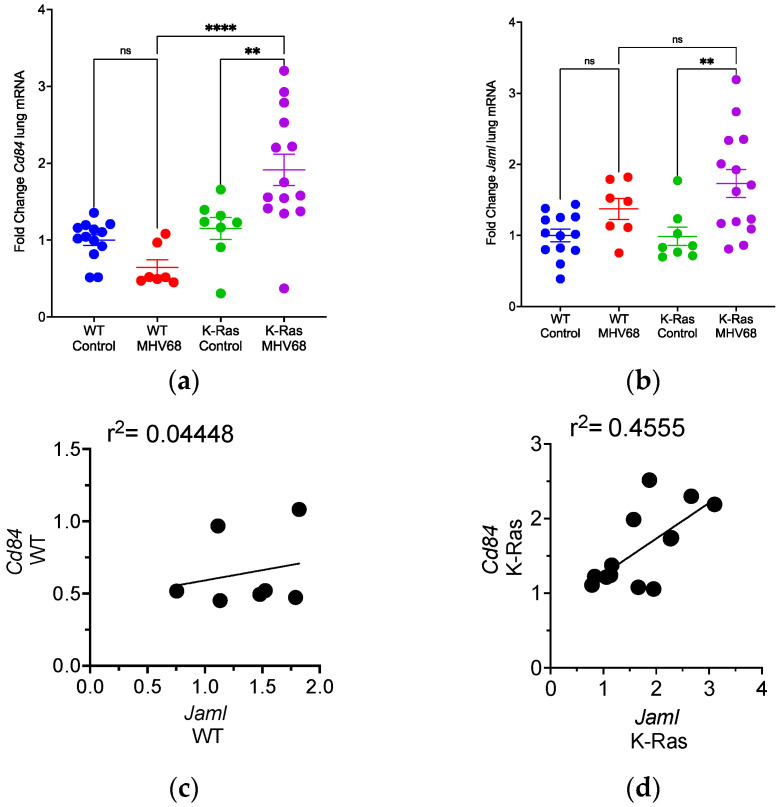
Differential expression of immune cell mRNA markers in the lungs of MHV68-infected wild-type and tumor-bearing mice. (**a**) Four-way comparison of mRNAs encoding immune cell markers between uninfected and infected wild-type and K-Ras^LA1^ mice at 7 days post-infection. Each symbol on the graph depicts the relative levels of *Cd84* mRNA in the lungs of a mouse in one of four treatment groups: uninfected wild-type (WT Control), infected wild-type (WT MHV68), uninfected K-Ras^LA1^ (K-Ras Control), and infected K-Ras^LA1^ (K-Ras MHV68). Each value is normalized to the mean of the WT Control group. Statistical significance was determined by ordinary one-way ANOVA. (ns, nonspecific; ** *p* = 0.0018, **** *p* < 0.0001). (**b**) Same as panel (**a**) except the levels of *Jaml* mRNA are shown. (ns, nonspecific; ** *p* = 0.0022). (**c**) Correlation graph for *Cd84* mRNA levels versus *Jaml* mRNA levels in wild-type mice infected with MHV68 (r^2^ = 0.04448). (**d**) Same as panel (**c**) except levels of *Cd84* mRNA are correlated with levels of *Jaml* mRNA in infected K-Ras^LA1^ mice (r^2^ = 0.4555).

Tumor-bearing mice may respond differently to MHV68 infection than wild-type mice regarding immune cells and related cytokine profiles, and potential differences would be more pertinent to tumor development. K-Ras^LA1^ and wild-type mice were infected with 40,000 pfu of MHV68 or mock-infected and harvested 7 days post-infection, a time of peak expression of *Il-6, Csf3, and Cxcl1* mRNAs. *Cd84* mRNA did not increase in MHV68 infected wild-type mice (Figure 4a), but *Jaml* mRNA did (Figure 4b). In contrast, both *Cd84* and *Jaml* mRNAs increased in MHV68-infected K-Ras^LA1^ mice (Figure 4a,b). *Cd84* mRNA levels did not correlate with *Jaml* mRNA levels in wild-type mice after MHV68 infection (Figure 4c), but these two MDSC markers did correlate in MHV68-infected K-Ras^LA1^ mice (Figure 4d).

Sequence analysis of lung RNA from uninfected and infected K-Ras^LA1^ mice (Figure S4a) showed a strong correlation between *Cd84* and *Jaml* mRNA expression (r^2^ = 0.87). Moreover, the RNA seq analyses revealed that both *Cd84* and *Jaml* mRNA levels in uninfected and infected K-Ras^LA1^ mice correlated well (r^2^ = 0.77 and 0.64, respectively) with the mRNA level for Triggering Receptors Expressed on Myeloid cells 2 (*Trem2*) mRNA (Figure S4b,c), which is also expressed by MDSCs, including in lung cancer [25,38,39]. These observations suggest immune cells are differentially recruited to the lung in wild-type and lung-tumor-bearing mice after infection with MHV68.

**Figure 5:**
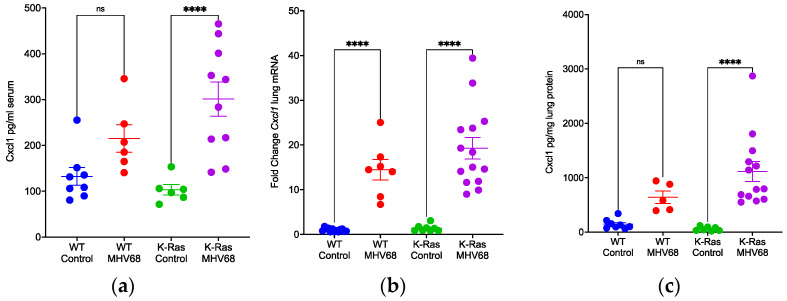
MHV68 infection selectively enhances expression of Cxcl1 protein in the lungs of tumor-bearing versus wild-type mice. Four-way comparison of Cxcl1 protein and mRNA expression between uninfected and infected wild-type and K-Ras^LA1^ mice at day 7 post-infection. (**a**) Serum levels (pg/mL) of Cxcl1 protein determined by ELISA in 4 groups of mice: uninfected wild-type (WT Control), infected wild-type (WT MHV68), uninfected K-Ras^LA1^ (K-Ras Control), and infected K-Ras^LA1^ (K-Ras MHV68) (ns, nonspecific; **** *p* < 0.0001). (**b**) Relative levels of *Cxcl1* mRNA in the lung. Same as Figure 4a except the relative levels of *Cxcl1* mRNA are shown. (**** *p* < 0.0001). (**c**) Same as panel (**a**) except pg Cxcl1 per mg lung extract is shown. (ns, nonspecific; **** *p* < 0.0001). Statistical comparisons by one way ANOVA.

Analyses of TCGA data to correlate prognosis with cytokine mRNA expression in lung adenocarcinoma patients identified increased levels of the mRNAs for *Csf3, Cxcl1*, and *IL-6* as significant indicators of a worse prognosis [40]. To better understand differential recruitment of immune cells to the lung between wild-type and K-Ras^LA1^ mice during MHV68 infection, we evaluated expression of Cxcl1 in the four experimental groups shown in Figure 4. *Cxcl1* lung mRNA increased 13.6-fold in the lungs of both wild-type and K-Ras^LA1^ mice after infection with MHV68 (Figure 5b). However, Cxcl1 expression at the protein level in the lung showed a greater than 18-fold (*p* < 0.0001, ANOVA) increase in infected K-Ras^LA1^ mice and much lower induction (4.3-fold, *p* = 0.057) in wild-type infected littermates (Figure 5c). After MHV68 infection, Cxcl1 protein levels in the serum increased 1.6-fold (*p* = 0.075 ANOVA) in wild-type mice and 2.9-fold (*p* < 0.0001, ANOVA) in K-Ras^LA1^ mice (Figure 5a). Thus, the differential infection-related induction of Cxcl1 protein levels in the lungs and sera of K-Ras^LA1^ mice relative to their wild-type littermates are consistent with differences in immune cell recruitment.

**Figure 6:**
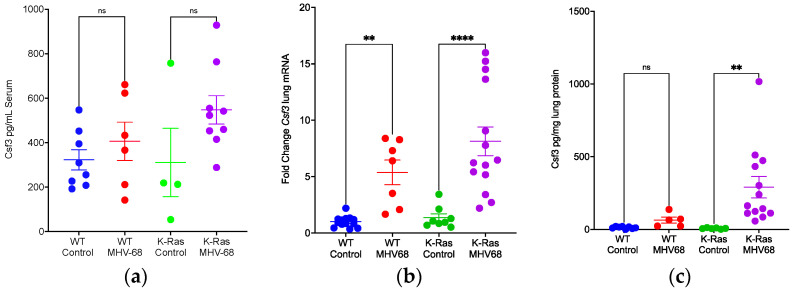
MHV68 infection selectively enhances expression of Csf3 protein in the lungs of tumor-bearing versus wild-type mice. Four-way comparison of Csf3 protein and mRNA expression between uninfected and infected wild-type and K-Ras^LA1^ mice at day 7 post-infection. (**a**) Same as Figure 5a, except serum levels (pg/mL) of Csf3 protein are shown. (ns, nonspecific). (**b**) Same as Figure 5b, except the lung levels of *Csf3* mRNA are shown (** *p* = 0.0042; **** *p <* 0.0001). (**c**) Same as panel (**a**) except pg Csf3 per mg lung extract is shown. (ns, nonspecific; ** *p* = 0.0015). Statistical comparisons by one way ANOVA (GraphPad Prism).

Similar analyses of Csf3 protein and mRNA expression revealed similar differences between wild-type and tumor-bearing mice after infection with MHV68. Csf3 levels in the serum were not significantly different between groups, but a potential modest increase (1.8-fold, *p* = 0.06, ANOVA) in infected tumor-bearing mice appeared to be reduced (1.3-fold, *p* = 0.45, ANOVA) in infected wild-type mice (Figure 6a). Both types of mice displayed infection-related increases (wild-type 5.4-fold, K-Ras^LA1^ 6-fold) in the levels of *Csf3* mRNA in the lung (Figure 6b). Sharp differences appeared in the levels of Csf3 protein in the lung, which increased 49-fold (*p* = 0.0015, ANOVA) post-infection in K-Ras^LA1^ mice relative to a 5-fold increase (*p* = 0.61, ANOVA) in mock-infected wild-type mice (Figure 6c). These observations may contribute to an altered immune environment in the lungs of K-Ras^LA1^ mice post-infection relative to that in the infected wild-type mice. Since Csf3 participates in MDSC recruitment and tumor promotion in multiple tumor models [34,41,42,43], this difference may contribute to the observations with MHV68-infected tumor-bearing mice shown here. The sequencing of lung RNA from infected versus uninfected K-Ras^LA1^ mice revealed increases in transcripts encoding immunosuppressive mediators (Figure S5), such as arginase 1 (*Arg1*, 6.6-fold), programmed death-ligand 1 (*Pd-l1/cd274*, ~20-fold), nitric oxide synthase 2 (*Nos2*, ~2.8-fold), and indoleamine 2,3-dioxygenase 1 (*Ido1*, ~110-fold). This observation is consistent with a tumor-promoting immune suppressive environment in the lung established by MHV68 infection at day 7 post-infection.

### 3.5. IL-6 Induction and Localization in Tumor-Bearing Mice

**Figure 7:**
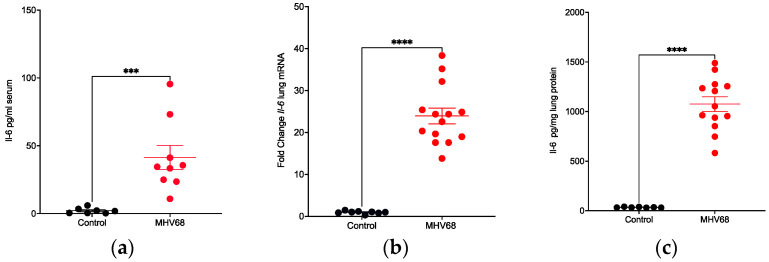
MHV68 infection induces IL-6 mRNA and protein expression in K-Ras^LA1^ mice. (**a**) Comparison of IL-6 protein levels in the serum of uninfected and MHV68-infected K-Ras^LA1^ mice. Each symbol represents the relative levels of IL-6 protein in the serum of an uninfected or MHV68-infected K-Ras^LA1^ mouse (*** *p* = 0.0002). (**b**) Comparison of *Il-6* mRNA levels in the lungs of uninfected and MHV68-infected K-Ras^LA1^ mice. Each symbol represents the relative levels of *Il-6* mRNA in the lungs of an uninfected or MHV68-infected K-Ras^LA1^ mouse (**** *p <* 0.0001 unexposed versus MHV68). (**c**) Comparison of Il-6 protein levels in the lungs of uninfected and MHV68-infected K-Ras^LA1^ mice. Each symbol represents the pg of IL-6 protein per mg lung extract from an uninfected or MHV68-infected mouse (**** *p <* 0.0001).

Since elevated levels of IL-6 in MHV68-infected wild-type mice were confirmed, the next question to address was how IL-6 would respond to infection in lung-tumor-bearing mice. MHV68 infection increased IL-6 protein levels in the serum of K-Ras^LA1^ mice by about 19-fold relative to mock-infected control mice (Figure 7a). Similarly, the mRNA levels of *Il-6* in the lung increased 25-fold relative to control (Figure 7b). The protein levels of IL-6 in lung tissue increased 31-fold (Figure 7c). Thus, MHV68 infection greatly enhanced both the mRNA (lung) and protein levels (lung, serum) of IL-6 in tumor-bearing mice.

**Figure 8:**
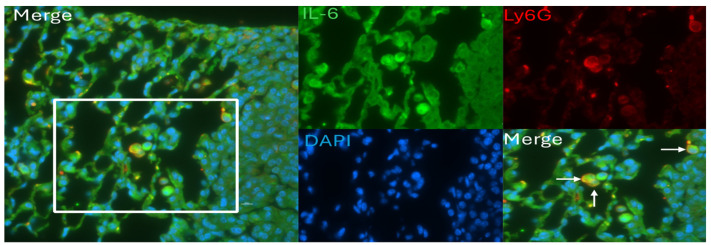
Ly6G/IL-6 double-positive cells in the lungs of MHV68-infected tumor-bearing mice. Formalin-fixed paraffin-embedded lung tissue sections from MHV68-infected K-Ras^LA1^ mice were co-stained with antibodies against IL-6 (green) and Ly6G (red) by immunofluorescence. The nuclei were visualized (blue) by staining with 4′,6-diamidino-2-phenylindole (DAPI). Detection of Ly6G/IL-6 double-positive cells at 600× magnification (Merge, arrows, lower right panel). The white bar to the right of the white square area of interest in the left Merge panel is equal to 10 microns. The densely packed cells on the right side of each panel are primarily lung tumor cells.

Gr-1^+^ MDSCs are a source of IL-6 in a murine colon cancer model [44]. Since the cytokines (Cxcl1 and Csf3) involved in immune cell recruitment and survival and *Cd84* are elevated at day 7 in tumor-bearing mice, we tested if Ly6G^+^ mononuclear cells are present in MHV68-infected mice and are also a source of IL-6. Lung tissue sections from MHV68-infected K-Ras^LA1^ mice at day 7 post-infection co-stained positively for both Ly6G and IL-6 (white arrows, Figure 8). Thus, it seems likely that recruitment of these cells at day 7 post-infection with MHV68 contributes to the increase in IL-6 at this time point.

### 3.6. Evaluation of Immune Cells in MHV68-Infected Tumor-Bearing Mice

**Figure 9:**
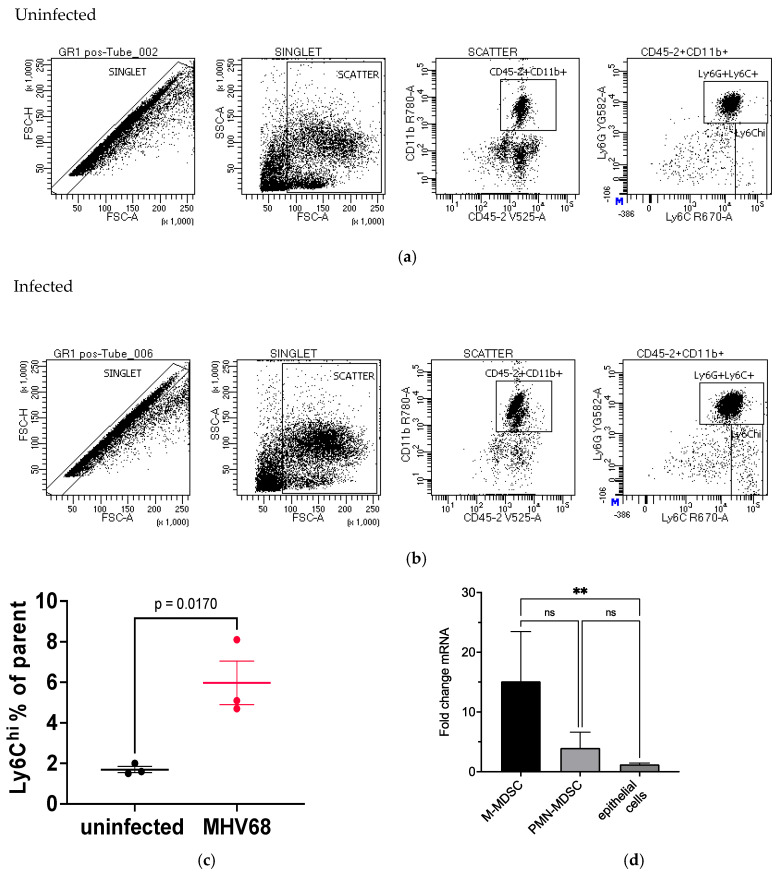
Detection of Ly6G^+^ cells in MHV68-infected tumor-bearing mice. Flow cytometry was used to analyze Ly6G^+^ cells from uninfected and MHV68-infected K-Ras^LA1^ mice 7days post-infection. Microbead-selected Ly6G^+^ cells were stained with CD45.2, CD11b, Ly6G, and Ly6C antibodies. (**a**) Representative flow cytometry data of Ly6G^+^/Ly6C^+^ cells from uninfected K-Ras^LA1^mice. (**b**) Representative flow cytometry data from Ly6G ^+^ cells from MHV68-infected K-Ras^LA1^ mice. (**c**) The graph represents the mean percentage of Ly6G^Dim^/Ly6C^hi^ cells relative to the input for the respective samples. Statistical comparison by unpaired t-test (GraphPad Prism). (**d**) The graph shows the fold change of selected marker mRNAs (Table S2) at day 7 after MHV68 infection relative to uninfected control K-Ras^LA1^ mice. mRNAs representative of M-MDSCs, but not PMN-MDSCs, increased relative to epithelial cell marker mRNAs after MHV68 infection of K-Ras^LA1^ mice (** *p* = 0.0047).

The preceding section describes immune cell recruitment and localization to the lung tumor microenvironment after MHV68 infection. We assessed Ly6G^+^ immune cells at day 7 after MHV68 infection of K-Ras^LA1^ mice by flow cytometry. First, Ly6G^+^ cells were selected twice by magnetic bead capture from a cell suspension of lung tissue from uninfected and MHV68-infected K-Ras^LA1^ mice. Then, the Ly6G^+^ cells were sorted by flow cytometry for CD45.2 and CD11b. The CD45.2/CD11b-enriched cells were then flow sorted with antibodies to Ly6G and Ly6C. The results showed very good Gr-1 enrichment, where Gr-1^+^ cells had a high content of Ly6G and Ly6C positive cells; however, we did not observe differences between MHV68-infected and uninfected tumor-bearing mice (Figure 9a,b). Further examination of the isolated Gr-1^+^ cells revealed differences in Ly6G^Dim^Ly6C^hi^ cell numbers between the MHV68-infected mice versus the PBS-treated controls. MHV68 infection of K-Ras^LA1^ mice induced selective recruitment of Ly6G^Dim^Ly6C^hi^ cells relative to the uninfected K-Ras^LA1^ mice (Figure 9c and Table S1). Ly6G^Dim^Ly6C^hi^ is a phenotypic marker for M-MDSCs, which are derived from progenitor monocytes [35]. An infection-related influx of immune cells was supported by RNA sequencing data that showed marker mRNAs for M-MDSCs increased ~4-fold when compared to marker mRNAs for PMN-MDSCs and increased 15.1-fold when compared to lung epithelial (alveolar type 1 and 2) marker mRNAs (Figure 9d and Table S2). The results suggest that the MDSCs are being selectively recruited during MHV68 infection, with an inclination towards M-MDSCs. In accordance with this assessment, RNA seq analysis of lung mRNA from MHV68-infected K-Ras^LA1^ mice revealed that *Ly6c1* and *Ly6c2* mRNAs, which encode the Ly6C surface marker, increased 3.1- and 10.1-fold, respectively, relative to their levels in lung mRNA from uninfected control K-Ras^LA1^ mice (Figure S6). This observation agrees with the selective recruitment of M-MDSCs to the lung in response to MHV68 infection of tumor-bearing mice, but functional analysis of these cells will be required to confirm this conclusion.

### 3.7. Differential m^6^A Methylation of Immune Cell Selective mRNAs in MHV68-Infected Tumor-Bearing Mice

**Figure 10:**
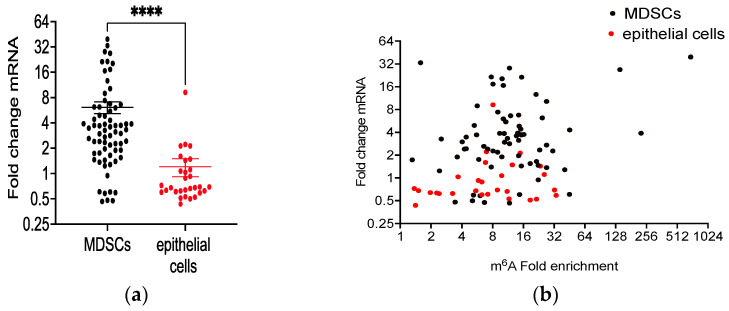
Differential expression of m^6^A-modified immune cell selective mRNAs in MHV68-infected tumor-bearing mice. (**a**) Post-infection m^6^A modification selectively enhances mRNA levels in MDSCs from infected K-Ras^LA1^mice. The graph shows the fold change in K-Ras^LA1^ mice at day 7 post-infection relative to the uninfected controls of m^6^A-modified marker mRNAs for MDSCs (black) and epithelial cells (AT1 and AT2 cells, red). (**** *p <* 0.0001 uninfected versus MHV68). (**b**) The graph shows the fold change for each m^6^A-modified mRNA versus m^6^A fold enrichment in the pull-down assay for marker mRNAs for MDSCs (black circles) and epithelial (AT1 and AT2) cells (red circles).

Promotion of autoimmune glomerulonephritis by IL-17 is mediated, in part, by m^6^A modification of RNA and functional IGF2BP2 (IMP2) binding of selected transcripts: *Cebpd, Ccl7, Mt2, Il-6,* and *Cxcl1* [33]. The expression level of each of these five transcripts in lung RNA was significantly enhanced by MHV68 infection of K-Ras^LA1^ mice (Figure S7). However, RNA-seq analyses of lung RNA isolated via an anti-m^6^A pull-down from infected tumor-bearing mice revealed significant m^6^A modification of only the transcripts for *Cebpd* and *Mt2*. To gain further insight into the relationship between m^6^A RNA modification and tumor promotion in response MHV68 infection, mRNAs identified previously for selective expression in MDSCs [45] were assessed for m^6^A RNA modification by the pull-down assay. Methylation of 69 MDSC marker mRNAs (Table S3) correlated with a greater than 6fold increase in expression following MHV68 infection (Figure 10a). In comparison, m^6^A-modified marker mRNAs for epithelial (alveolar type 1, AT1 and alveolar type 2, AT2, Table S3) cells [46] only showed a modest 1.2-fold increase (Figure 10a). This difference did not appear to be related to an elevated fold enrichment of m^6^A targets in MDSCs (Figure 10b). These data suggest that MHV68 infection induced selective m^6^A modification of MDSC markers and these preferentially methylated transcripts showed increased expression levels. Whether m^6^A modification is integral to the lung recruitment of MDSCs and the functional validation of these cells from MHV68-infected lung-tumor-bearing mice will require additional investigation.

## 4. Discussion

The results showed that MHV68 infection in tumor-bearing K-Ras^LA1^ mice increased the levels of Type 17 cytokines and proliferative lesions in the lung. The combination of virus infection and lung tumors correlated with the recruitment of immune cells to the lung. Although the presence of lung tumors was sufficient to promote lung recruitment of Gr-1^+^ cells, MHV68 infection led to recruitment of ^-^Ly6C^hi^ cells characteristic of M-MDSCs. In agreement with the recent observation regarding the recruitment of MDSCs (primarily Ly6C^hi^ cells) following ionizing radiation [47], m^6^A modification of RNA appears to contribute to enhanced levels of mRNAs selectively expressed in MDSCs. These observations support the premise that γherpesvirus infection establishes an immune environment that facilitates lung tumorigenesis and identifies the m^6^A modification of mRNA as a contributor to that process.

IL-17 expression promotes tumorigenesis in autochthonous murine lung tumor models [4,5,6,48]. Our assays of wild-type and tumor-bearing mice showed that MHV68 infection induced Type 17 inflammation, with a marked induction of IL-6. Promotion of lung tumorigenesis by Type 17 inflammation can be abrogated by an antibody to Gr-1 [5,6], and we identified Type 17 cytokines and Gr-1^+^ cells in MHV68-infected mice. MDSCs produce IL-6 [44], and the diminishing levels of Gr-1^+^ cells likely reduce tumor promotion, in part through the reduction of IL-6 levels. It is notable that Kaposi’s sarcoma-associated herpesvirus, a γherpesvirus that causes a human malignancy derived from endothelial cells, encodes a viral gene with homology to *Il-6* [11].

The induction of IL-6 and CSF3 in MHV68-infected K-Ras^LA1^ mice implicates tumor promotion mediated by MDSC recruitment. Further study will be required to functionally validate these cells as M-MDSCs. Since the literature has demonstrated that IL-17, IL-6, and CSF3 promote lung tumorigenesis [4,5,6,41,49,50], we conjecture that MHV68 induces tumor promotion via induction of these cytokines. However, future experiments will be required to verify that CSF3 and IL-6 are involved in tumor promotion in this model. Corresponding to the observations here, conversion of slowly cycling tumor cells in culture to a highly proliferative and invasive phenotype requires the stromal synthesis of both CSF3 and IL-6 [51]. Stromal fibroblasts release IL-6, CSF3, and Activin-A, which promote the dedifferentiation of lung carcinoma cells to cancer stem cells [52]. Treatment of tumor stromal organoids in culture with docetaxel induces cytokine secretion (Csf3, Csf2, Il-6, Cxcl1, Cxcl2, and Tnfα) into the cell culture media while reducing levels of Vegf [51]. Our RNA sequencing data revealed that similar alterations of the mRNAs encoding these cytokines occurred in the lungs of MHV68-infected K-Ras^LA1^ mice (with the exception of *csf2*, which did not change, Figure S8). In tumor-bearing mice, the combination of CSF3 and IL-6 cooperates to convert neutrophil precursors in the bone marrow into a pro-tumorigenic phenotype, while bone-marrow-derived neutrophils from mice lacking tumors have an anti-tumorigenic effect [53]. Consistent with this observation, the data shown here imply that the recruitment of immune cells (potentially MDSCs) depends on the combined effects of inflammation associated with viral infection and the presence of lung tumors, which correlates with increased expression of both IL-6 and CSF3. In multiple tumor models, tumor promotion by IL-17, IL-6, and CSF3 depends, at least in part, upon accumulation of MDSCs [5,6,34,35]. Co-staining demonstrated the presence of Ly6G and IL-6 double-positive cells in lung tissue sections from MHV68-infected K-Ras^LA1^ mice 7-days post-infection. These results are consistent with our findings, which show that MHV68 infection enhanced both the mRNA (lung) and protein levels (lung, serum) of IL-6 in tumor-bearing mice 7-days post-infection, a time when MDSC-promoting cytokines (Csf3 and Cxcl1) and MDSC markers (*Cd84*, *Jaml*, and *Trem2*) were also significantly elevated. These results are further supported by the literature, which attests that Gr-1^+^ MDSCs are a source of IL-6 [44] and that IL-6 has a significant role in tumor promotion [54,55,56,57,58]. A cytokine with homology to IL-6 promotes invasion/metastasis via a feed-forward loop in a drosophila model [59]. In colon carcinoma, IL-6 is expressed by MDSCs in the tumor microenvironment and tumor promotion is abrogated upon ablation of IL-6 expression and enhanced by IL-6 overexpression [44]. IL-6 has pleiotropic effects on immune cells, which contributes to its role in tumor promotion and as a regulator of MDSC activity [49]. It also plays a role in tumor cell proliferation, survival, invasiveness, and metastasis [54,55,56,57,58].

In this model of lung tumor promotion by MHV68, induction of Csf3 requires both the infection and the presence of lung tumors. Csf3 mediates MDSC promotion [34] and chemotherapy resistance [51]. Elevated plasma levels of Csf3 are a poor prognostic indicator in non-small cell lung cancer [50]. Csf3 promotes the survival, proliferation, differentiation, and function of mature neutrophils and neutrophil precursors [34]. Csf3 and Cxcl1, which are induced by IL-17 in our lung tumor cell line from K-Ras^LA1^ mice, contribute to the development, function, and recruitment of MDSCs [34,35]. The enhanced expression of *Cd84*, *Jaml*, and *Trem2* mRNAs in MHV68-infected mice conform to MDSC accumulation in the lung. In mice injected with colon cancer cells, Csf3 promotes tumorigenesis by eliciting IL-10-producing FoxP3^+^ CD4^+^ and CD8^+^ T cells, and [60] mice lacking Csf3r display reduced levels of IL-10 and decreased growth of injected tumor cells [60]. In accord, we observed increased *Il-10*, *Cd4*, *Cd8a*, and *Foxp3* mRNAs in our RNA-seq data from K-Ras^LA1^ mice on day 7 post-infection (Figure S9). A significant difference in lung Csf3 and Cxcl1 protein levels between MHV68-infected wild-type and tumor-bearing mice was observed and appears to correlate with the differential lung recruitment of MDSCs as assessed by mRNA levels of *Cd84*, *Jaml*, and *Trem2* expression in the lung and detection of CD45^+^CD11b^+^Ly6G^+^ and CD45^+^CD11b^+^Ly6G^Dim^Ly6C^+^ cells by flow cytometry.

MDSCs are a group of highly heterogeneous cells derived from immature myeloid progenitors that are usually divided into two subpopulations: polymorphonuclear MDSCs (PMN-MDSCs) and monocytic MDSCs (M-MDSCs) [61]. During tumor development, MDSCs are recruited to the tumor site by chemokines, where they induce tumor promotion by expressing IL-6 and suppressing T- and NK-cell activation [25,34,35,44,62]. Lower MDSC levels correlate with prolonged patient survival in glioblastoma and glioma [47]. PMN-MDSCs have been typically associated with tumor promotion by IL-17 [34]. At 7 days post-infection, there was more recruitment of Ly6C^hi^ cells in the Ly6G^+^-isolated samples from MHV68-infected K-Ras^LA1^ mice than there were in the uninfected K-Ras^LA1^ mice. Since Ly6C^hi^ is a phenotypic marker for M-MDSCs, these data imply these cells derived from progenitor monocytes are being induced by MHV68 infection. These Ly6C^hi^ cells effectively suppress T cells [63], and Trem2 expression by M-MDSCs appears to play an essential role in the suppression of NK cells in lung cancer [25]. Potentially, the CCL2-CCR2 ligand–receptor pair are involved in recruitment of these Ly6G^-^/Ly6C^hi^ cells to the lung. CCR2^+^ M-MDSCs suppress CD8 T cells in a murine melanoma model [64]. The enhanced detection of M-MDSC markers at day 7 post-infection (Figure 9c) indicates that these cells are being selectively recruited during MHV68 infection.

The most prevalent eukaryotic mRNA modification is N^6^-methyladenosine (m^6^A), and it is responsible for regulating the translation and stability of modified mRNAs [65]. RNA methylation appears to correlate with the enhanced expression of mRNAs selectively expressed in MDSCs (Figure 10). This correlation suggests that therapeutic targeting of RNA modification may provide a means to reverse mobilization of immune cells to the lung and thereby ameliorate γherpesvirus-dependent immune suppression in lung tumorigenesis. Our data describe an altered immune milieu during lung tumorigenesis associated with γherpesvirus infection.

## Figures and Tables

**Table 1:** Primers used for qPCR.

Primer	Forward Sequence	Reverse Sequence
ORF65	5′GTCAGGGCCCAGTCCGTA3′	5′TGGCCCTCTACCTTCTGTTGA3′
IL-17A	5′CAGACTACCTCAACCGTTCCAC3′	5′TCCAGCTTTCCCTCCGCATTGA3′
IL-6	5′TACCACTTCACAAGTCGGAGGC3′	5′CTGCAAGTGCATCATCGTTGTT3′
CXCL1	5′CTGGGATTCACCTCAAGAACATC3′	5′CAGGGTCAAGGCAAGCCTC3′
CSF3	5′ATGGCTCAACTTTCTGCCCAG3′	5′CTGACAGTGACCAGGGGAAC3′
CD84	5′ATATAGCTGGAGTCCCTTTGGAG3′	5′AAAGAGCACGGCCAATCCTC3′
JAML	5′ATGCTTTGCCTCCTGAAACTG3′	5′TGATTCACCCACATGCACTCT3′
ß-actin	5′GATGTATGAAAGCTTTGGTC3′	5′TGTGCACTTTTATTGGTCTC3′

**Table 2:** Antibodies used for immunofluorescence and flow cytometry.

Antibody	Detection	Source and Catalog Number
Purified Rat Anti-Mouse Ly6G (1A8)	Ly6G	BD Pharmingen cat#: 551459/clone1A8
Rabbit IL-6 Antibody	IL-6	Novus Biologicals cat#: NB600-1131
Goat Anti-Rat IgG H&L (Alexa Fluor 647)	Secondary anti-rat	Abcam cat#: ab150159
Goat Anti-Rabbit IgG H&L (Alexa Fluor 488)	Secondary anti-rabbit	Abcam cat#: ab150077
Rat APC-eFluor780 CD11b	CD11b	Invitrogen cat#: 47-0112-80/clone M1/70
Rat APC Anti-Mouse Ly6C	Ly6C	BioLegend cat#: 128015/clone HK1.4
Mouse V500 Mouse Anti-Mouse CD45.2	CD45.2	BD Horizon cat#: 562130/clone 104
PE Rat Anti-Mouse Ly6G (1A8)	Ly6G	BD Pharmingen/cat#: 561104/clone 1A8

## Data Availability

The original contributions presented in the study are included in the article/supplementary material, further inquiries can be directed to the corresponding author.

## Supplementary Materials

The following supporting information can be downloaded at: https://www.mdpi.com/article/10.3390/pathogens13090747/s1. Figure S1: MHV68 infection induces Il-6 protein expression in wild-type mice. Figure S2: IL-17 treatment of mK-Ras-LE cells induces mRNAs encoding cytokines that promote granulocyte recruitment and survival. Figure S3: Changes in *Jaml* mRNA levels at increasing times after infection of wild-type mice. Figure S4: MDSC marker correlations observed in the lungs of uninfected and MHV68-infected tumor-bearing mice. Figure S5: Induction of immunosuppressive mediators in MHV68-infected tumor-bearing mice. Figure S6: Increased expression of mRNAs encoding M-MDSC surface markers after infection with MHV68. Figure S7: MHV68-mediated induction of lung levels of mRNAs identified previously as m^6^A modified in an IL-17-dependent manner [33]. Figure S8: Profiling inflammatory cytokine mRNA expression in MHV68-infected tumor-bearing mice. Figure S9: mRNA markers of suppressed adaptive immunity in MHV68-infected mice one-week post-infection. Table S1: Analyses of Ly6G^+^ by flow cytometry. Table S2: mRNAs selectively expressed in M-MDSCs, PMN-MDSCs, Alveolar Type 1 (AT1) cells, and Alveolar Type 2 (AT2) cells. Table S3: Fold-change mRNA vs fold-m^6^A-enrichment for MDSC versus lung epithelium marker mRNAs.
