# Supplementary material for: Gammaherpesvirus Infection Stimulates Lung Tumor-Promoting Inflammation
Source: Pathogens. 2024 Aug 31;13(9):747. doi: 10.3390/pathogens13090747 (PMC11434807; doi:10.3390/pathogens13090747)
Supplement: Supplementary file 1 [file pathogens-13-00747-s001.zip › Mukhopadhyay Pathogens supplemental data.pdf]

pathogens

MDPI

Supplemental Data

Gammaherpesvirus infection stimulates lung tumor-promoting inflammation

Sudurika S Mukhopadhyay, Kenneth F Swan, Gabriella Pridjian, Jay K Kolls, Yan Zhuang, Qinyan Yin, Joseph A Lasky, Erik Flemington, Cindy A Morris, Zhen Lin, Gilbert F Morris

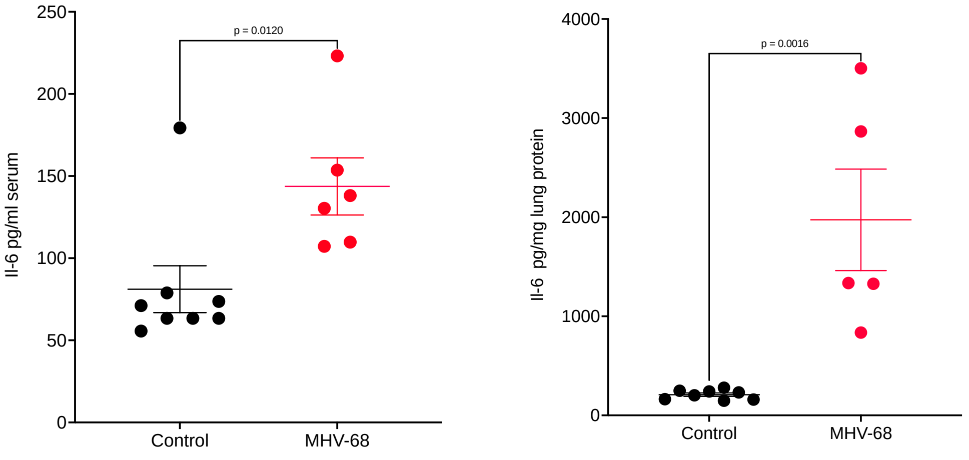

(a) **(b)**

Figure S1. MHV68 infection induces IL-6 protein expression in WT mice. **(a)** Comparison of IL-6 protein levels in the serum determined by ELISA of uninfected and MHV68 infected WT mice. Each symbol represents the relative levels of IL-6 protein in the serum of an uninfected or MHV68 infected WT mouse (*p=0.0120) **(b)**. Comparison of IL-6 protein levels in the lungs of uninfected and MHV68 infected WT mice. Each symbol represents the pg of IL-6 protein per mg lung extract from an uninfected or MHV68 infected WT mouse (**p=0.0016).

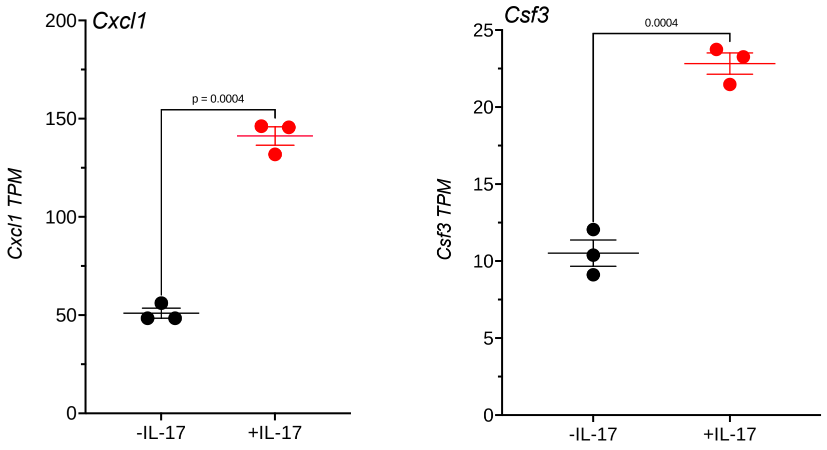

(a) **(b)**

Figure S2. IL-17 treated mK-Ras-LE cells induce mRNAs encoding cytokines that promote granulocyte recruitment and survival. mK-Ras-LE cells were placed in serum-free media for 24 hrs prior to changing the serum-free media with and without 10ng/ml IL-17A. After an additional 24 hrs, RNA was prepared from the cells and submitted for sequencing. RNA sequencing analysis

revealed increased expression of key mRNAs encoding proteins involved in MDSC recruitment. **(a)** *cxcl1* **(b)** *csf3*. Significance determined by Welch's t-test (GraphPad Prism).

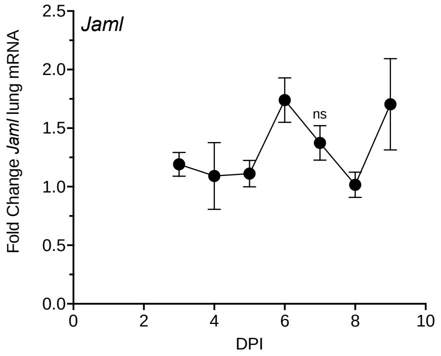

Figure S3. MHV68 infection of wild-type mice does not affect expression of *jaml*, a MDSC marker mRNA. Levels of *jaml* mRNA in the lungs of wild-type mice were determined at increasing times post-infection with MHV68 (see Figure 1). The graph shows lung mRNA expression levels for *jaml* for the indicated day post-infection (DPI) showing no significant change 7-days post-infection. (ns, nonspecific; infected versus uninfected control, (n=3-9; n=13 control).

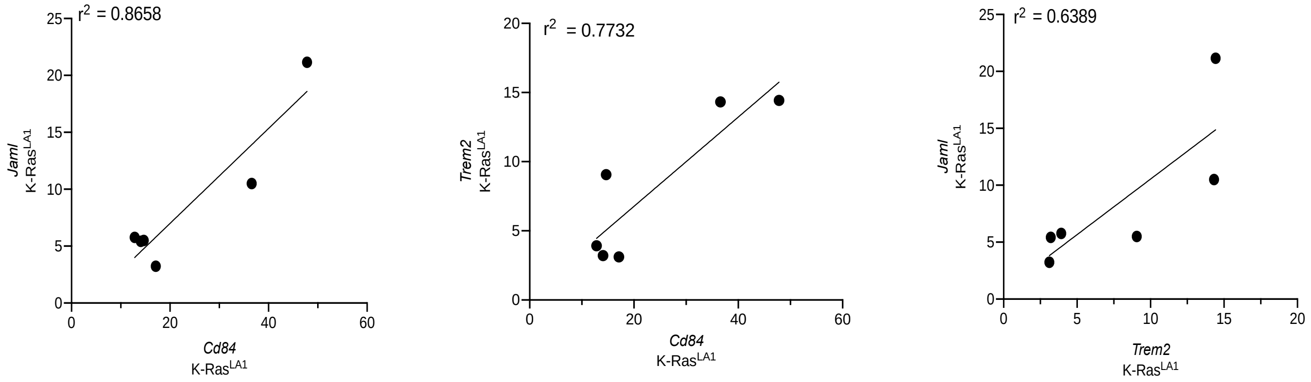

(a) (b) (c)

Figure S4. MDSC marker correlations observed in the lungs of uninfected and MHV68-infected tumor-bearing mice. **(a)** Correlation graph from RNA seq analyses of lung mRNA for *cd84* versus *jaml* in uninfected and infected K-Ras^{LA1} mice at 7 days post-infection (r^2=0.8658). **(b)** Same as panel a except levels of *cd84* mRNA are correlated with levels of *trem2* mRNA (r^2=0.7732). **(c)** Same as panel a except levels of *jaml* mRNA are correlated with levels of *trem2* mRNA (r^2=0.6389).

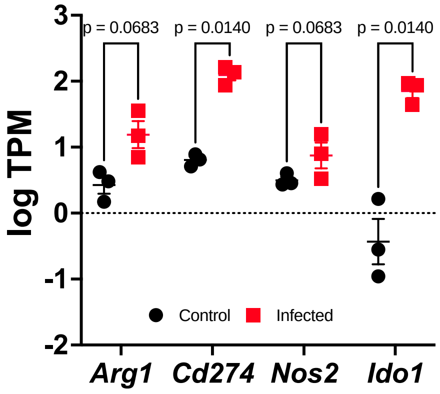

Figure S5. Induction of immunosuppressive mediators in MHV68-infected tumor-bearing mice at day 7 post-infection. RNA sequencing analysis of lung RNA from uninfected versus infected K-Ras^{LA1} mice 7-days post-infection implicated induction of transcripts encoding the indicated immunosuppressive mediators. Transcripts per million (TPM) values for each mRNA were log

transformed. Uninfected K-Ras^{LA1} mice (black circles); MHV68 infected K-Ras^{LA1} mice (red squares). Significance determined by multiple paired t-test (GraphPad Prism).

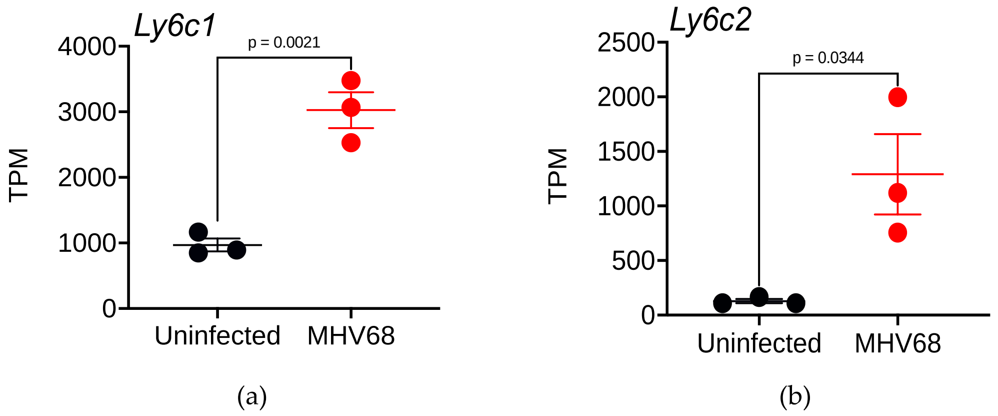

Figure S6. Promotion of M-MDSC surface marker post MHV68-infection. RNA sequencing analysis of lung mRNA revealed increased *ly6c* mRNAs encoding the Ly6c surface marker in MHV68 infected K-Ras^{LA1} mice 7-days post-infection when compared to their respective uninfected K-Ras^{LA1} controls. Uninfected (black circles); MHV68 infected (red circles). **(a)** *ly6c1* **(b)** *ly6c2*. Significance determined by unpaired t-test (GraphPad Prism).

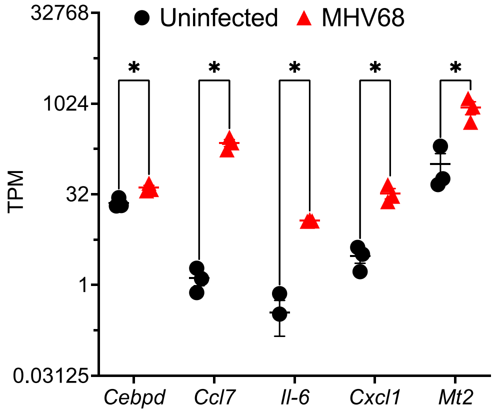

Figure S7. Selected IL-17-targeted transcripts [27] are increased post MHV68-infection. RNA sequencing analysis of lung mRNA revealed increased levels of the indicated transcripts in MHV68-infected K-Ras^{LA1} mice 7-days post-infection. Uninfected (black circles); MHV68 infected (red triangles). *$p<0.05$, uninfected versus MHV68-infected (multiple paired t-test, GraphPad Prism).

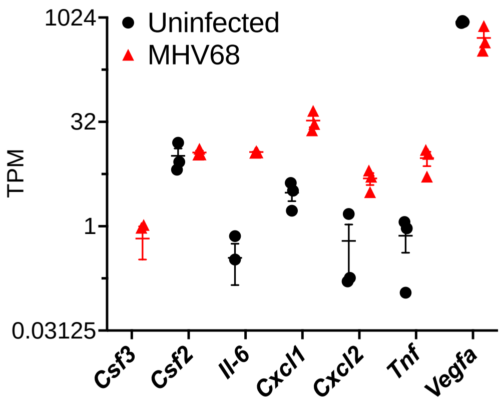

Figure S8. MHV68 infection enhances inflammatory cytokine mRNA expression in tumor-bearing mice. RNA sequencing data revealed induction of inflammatory cytokine mRNAs *Csf3*, *Il-6*, *Cxcl1*, *Cxcl2*, and *Tnf* in the lungs of MHV68 infected K-Ras^{LA1} mice

7-days post-infection. mRNA levels of *csf2* did not change whereas *vegfa* mRNA was reduced. Uninfected (black circles); MHV68 infected (red triangles).

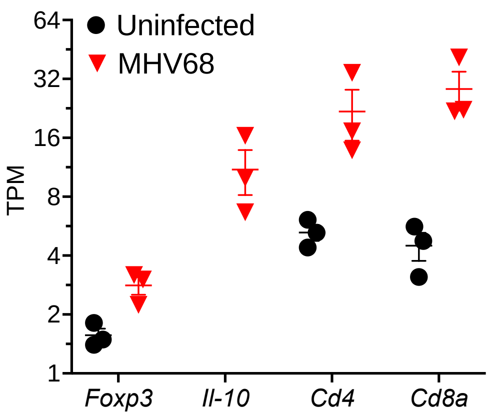

Figure S9. mRNA markers suggest suppressed adaptive immunity in MHV68 infected mice at one week post-infection. RNA sequencing analysis of lung RNA revealed increased *foxp3*, *il-10*, *cd4*, and *cd8a* mRNAs in MHV68 infected K-Ras^{LA1} mice 7-days post-infection when compared to their respective uninfected K-Ras^{LA1} controls. Uninfected (black circles); MHV68 infected (red triangles).
